# Mpox in UK households: estimating secondary attack rates and factors associated with transmission, May–November 2022

**DOI:** 10.1017/S0950268824000864

**Published:** 2024-10-02

**Authors:** Simon Packer, Piotr Patrzylas, Rachel Merrick, Clare Sawyer, Andrew McAuley, William Crowe, Gillian Armstrong, Leonardo Green, Lucy Findlater, Charlie Turner, Obaghe Edeghere, Charlotte Anderson

**Affiliations:** 1 UK Health Security Agency, London, UK; 2 UK Field Epidemiology Training Programme (UKFETP), UK Health Security Agency, London, UK; 3 Communicable Disease Surveillance Centre, Public Health Wales, Cardiff, UK; 4 Public Health Scotland, Edinburgh, UK; 5 School of Health and Life Sciences, Glasgow Caledonian University, Glasgow, UK; 6 Public Health Agency, Belfast, UK

**Keywords:** Mpox, MPXV, Epidemiology, Transmission, Contact, Outbreak

## Abstract

We aimed to estimate the secondary attack rate of mpox among UK household contacts and determine factors associated with transmission to inform public health management of contacts, during the global outbreak in 2022. Information was collected via NHS and public health services and included age, gender, place of residence, setting, and type of contact. Aggregate information was summarized for the UK. Record level data was combined for England, Wales and Northern Ireland, and multivariable logistic regression was used to determine factors associated with transmission. The secondary attack rate among UK household mpox contacts was 4% (60/1 526). Sexual contact with the index case was associated with a 11-fold increase in adjusted odds of becoming a case in England, Wales, and Northern Ireland (95% CI 5.5–22, *p* < 0.001). Household contacts outside of London had increased odds compared to London residents (adjusted OR 2.9, 95%CI 1.6–5.4, *p* < 0.001), while female contacts had reduced odds of becoming a case (aOR: 0.41, 95% CI: 0.15–0.95). We found a low overall secondary attack rate among household mpox contacts with strong evidence of increased transmission risk associated with sexual contact. This evidence will inform the risk assessment of contacts and support prioritization of those with close intimate contact for follow up.

## Introduction

Human mpox disease, a viral zoonotic infection with the monkeypox virus (MPXV), endemic to West and Central Africa was first detected in the Democratic Republic of Congo in the 1970s [1, 2]. Before 2022, the global epidemiology of mpox was characterized as a largely sporadic, predominantly zoonotic disease occurring in West and Central Africa, with a minority of cases occurring from human–human transmission [1]. In the United Kingdom (UK) mpox was rarely seen, with only seven human cases between 2018 and 2021. Four of these UK cases were defined as importation events from endemic countries and three were due to domestic transmission with no known wider community spread [3].

Person to person transmission of MPXV requires close contact with respiratory secretions, skin lesions or contaminated objects [2]. Previous studies have identified immediate family and household contacts as those at greatest risk of onward MPXV transmission [4]. Household members have an increased risk of sustained close contact with skin lesions and contaminated objects, for instance when caring for infected persons [5]. A systematic review found a secondary attack rate of 5% in household contacts of an infectious case of mpox [4], though this was based on just four papers describing cases in Zaire and the Democratic Republic of the Congo, between 1980 and 2012. Transmission chains were short with a low number of tertiary and quaternary cases identified [1, 3, 6].

In May 2022, a large community-based outbreak of mpox emerged in the UK and worldwide, and WHO declared mpox a Public Health Emergency of International Concern (PHEIC) on 23 July 2022 [7]. In contrast to previous incidents, cases were detected in people who had not travelled to endemic countries or had contact with a known case [7]. As of 14 November 2022, 3 710 confirmed or highly probable cases of mpox had been reported in the UK [7]. The majority (69%) were in London and 99% were men, with a median age of 37 years [7]. Cases primarily identified as gay, bisexual and other men who have sex with men (GBMSM) [7]. In contrast to previous outbreaks identified in the UK, direct physical contact between cases in the global outbreak was predominately sexual rather than care giving [4, 8, 9].

In addition to the clinical management of cases, the public health response to this outbreak included risk communication and community engagement, contact tracing and vaccination of at-risk groups.

It is currently unknown to what extent household exposure contributed to onward transmission in the UK during the global 2022 outbreak, particularly without sexual contact. We aimed to estimate the secondary attack rate of mpox among UK household contacts between May and November 2022 and identify factors associated with transmission to inform public health management of contacts.

## Methods

### Study design and population

A retrospective cohort study was undertaken to examine the association between household contact with a case and developing mpox.

The study included all eligible cases and associated contacts reported between 6 May and 24 November 2022 in the UK.

### Study definitions

A case refers to people meeting either the confirmed or highly probable case definition:

- A confirmed case was a UK resident with laboratory-confirmed MPXV infection (MPXV DNA positive on PCR), reported between 6 May and 24 November 2022 [10].
- A highly probable case was a UK resident with orthopox virus PCR positive result where mpox remained the most likely diagnosis [11], reported between 6 May and 24 November 2022.

Contacts were persons named by a case and were exposed to that case during their potentially infectious (symptomatic) period [12]. Household contacts were defined using data recorded by health protection professionals or sexual health clinicians on national case management systems. Household contacts had a principal contextual setting (setting where the majority of exposure to a case was thought to have taken place) defined as a ‘household setting’. If the principal contextual setting was unknown, exposure group (the type of contact that was experienced) was reviewed, and they were defined as a household contact if this was ‘household exposure’. Household contacts within institutional settings like care homes, hostels, or hotels were excluded.

Sexual contact was defined as where the degree of contact (type of contact with the case) with the person was recorded as ‘intimate contact’ (in England and Northern Ireland) or ‘sexual contact’ (in Wales). For cases in England, free text analysis was used to enhance contact classification. Free text fields were searched for words or phrases indicating sexual contact between index case and contact. Classifications were manually reviewed by the lead analytical authors to ensure accuracy.

### Outcome definition

An index case was a confirmed or highly probable case, with the date of symptom onset (if missing, used date of positive specimen or laboratory report date) on the same day or before a linked contact who was subsequently identified as a secondary case.

A secondary case was a confirmed or highly probable case, with the date of symptom onset (if missing, date of positive specimen or laboratory report date) on the same day or after the linked index case.

Index – secondary case pairs were classified based on the directionality of transmission, with the index case occurring before or on the same day of the secondary case. An index – secondary case pair could only appear once. When case pairs occurred on the same day, index and secondary case definitions were assigned arbitrarily.

### Inclusion and exclusion criteria

Duplicate case records were included to ensure that all associated contact records were included. Contacts associated with duplicate case records were checked for uniqueness. Contact records were excluded if they were marked as discarded or if they were already recorded as an index case (if a person was recorded as a case but was declared as a contact by another case, the contact record was removed). Contacts were unable to be deduplicated due to inconsistent and limited identifiers.

### Data collection method

In England, Wales, and Northern Ireland, information on cases and their associated contacts were collected by public health practitioners working in local health protection teams or sexual health clinicians. Data on contacts included age, gender, and setting of contact. In Wales, the date of last known contact with the case was also collected.

In Scotland, information on cases were collected through enhanced surveillance forms and held by Public Health Scotland. Contact tracing was done at the Health Board level with no data sharing with Public Health Scotland, therefore only aggregate information on household contacts was available, with no further detail on individual contacts.

### Data management

Data for cases and contacts in England were stored and accessed through a SQL server database. An analytical dataset was produced by extracting all cases and associated contacts and applying study definitions to obtain the eligible study population of interest. Secondary cases were identified by calculating the time difference (using symptom onset, or if missing, specimen or laboratory report date) between index and secondary cases to determine the directionality of pairs. If the index case had a corresponding contact record, these were removed.

Welsh data were held and managed by Public Health Wales. Data on cases and contacts were stored and accessed through a SQL server database. An analytical dataset comprising of cases and contacts linked to the Welsh case management system was extracted and anonymised. Data were shared with UKHSA for analysis using a secure data sharing platform.

The Northern Ireland Public Health Agency held and managed data on cases and contacts resident in Northern Ireland. Data on confirmed and pending laboratory results was held on the case management system (HPZone). Confirmed cases were interviewed and their contacts’ information was held on a SharePoint site. Data were shared securely with UKHSA for analysis.

Scottish data were held and managed by Public Health Scotland. As detailed data on contacts were not available, no data were shared for combined analysis purposes.

### Statistical methods

Characteristics, such as age, gender, residential location, and type of contact, of household contacts were described. Secondary attack rates were calculated by dividing the number of household contacts identified as secondary cases by the total number of household contacts. Secondary attack rates were stratified by month of index case identification, residential location, selected demographic characteristics, and sexual contact. Univariable logistic regression was undertaken to measure the association between different exposure types and a contact becoming a case. Odds ratios (OR) and corresponding 95% confidence intervals (95% CI) were calculated. Multivariable logistic regression was undertaken utilizing a forward stepwise approach with variables associated with secondary cases in univariable analysis (*p* < 0.1) added sequentially. Akaike information criterion (AIC) and likelihood ratio test statistics were examined to understand the variable contribution to the multivariable model. Adjusted OR and corresponding 95% CI were calculated using the final model. Statistical analysis was undertaken in R version 4.2.1 [13].

## Results

### England

**Figure 1.**
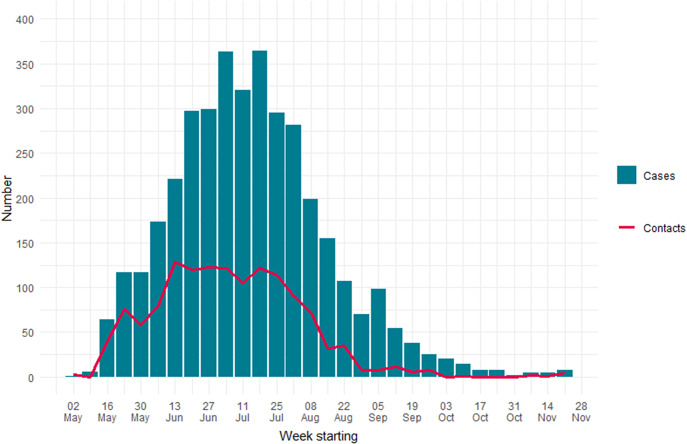
Number of confirmed or highly probable cases of mpox (bars, *n* = 3 734), and number of household contacts reported (line, *n* = 1 371), by week of report to UKHSA, England, 6 May to 24 November 2022.

From 6 May to 24 November 2022, there were 3 734 confirmed or highly probable cases of mpox in England, who reported 3 645 contacts: of these, 1 371 were household contacts (Figure 1). Twenty-three percent (314) of household contacts were known to have sexual contact with the index case. Nearly half of all household contacts were resident in London (634, 46%) and 69% were male (943). The median age was 36 years (IQR: 27–50 years, range: 0–85 years). For the 824 cases who reported at least one household contact, the average (mean) was 1.7 household contacts (an average total household size of 2.7 including the index case).

### Wales

The 47 mpox cases resident in Wales reported 137 contacts across all categories, including 29 household contacts. Five household contacts (17%) were classified as sexual contact. Household contacts had a median age of 40 years, and 54% were male. Of the 17 cases who reported at least one household contact, the mean was 1.7 household contacts (an average total household size of 2.7).

### Northern Ireland

There were 34 cases of mpox resident in Northern Ireland, who reported 57 contacts: of these, 28 were household contacts. Only 3 of 28 were known to have sexual contact. Household contacts had a median age of 60 years, 14 were female, and 14 were male. Of the 18 cases who reported at least one household contact, the mean was 1.8 household contacts (an average total household size of 2.8).

### Scotland

The 97 cases of mpox resident in Scotland reported 364 contacts across all settings, 98 of whom were household contacts. No further detail was available on the characteristics of these contacts.

### The UK (England, Wales, Scotland, and Northern Ireland)

The 3 912 UK cases of mpox reported 4 203 contacts between 6 May to 24 November 2022: 1 526 (36%) of these were defined as household contacts (Table 1). Sixty-two household contacts were known to subsequently become a case of mpox, an overall secondary attack rate of 4% to UK household contacts.

**Table 1.** Number of mpox cases, contacts and household contacts, and secondary attack rates among household contacts, by country of residence, 6 May to 24 November 2022, UK

				Secondary attack rate among household contacts	
	Total cases	Total contacts	Household contacts	*n*	%
England	3 734	3 645	1 371	55	4
Northern Ireland	34	57	28	0	0
Scotland	97	364	98	3	3
Wales	47	137	29	2	7
UK	3 912	4 203	1 526	60	4

### England, Wales, and Northern Ireland

As no further detail was available on the contacts in Scotland, further analysis was restricted to contacts resident in England, Wales, and Northern Ireland.

The 3 815 cases of mpox in England, Wales and Northern Ireland reported 1 428 household contacts. Age was unknown for 32% (463) of household contacts and gender for 2% (26). Among those with information, 68% (972) were male, and the most common age group was 26 to 35 years, followed by 36 to 45 years (Table 2).

**Table 2.** Descriptive and univariable analysis of characteristics associated with household mpox contacts becoming a case, England, Wales and Northern Ireland, 6 May to 24 November 2022

	Descriptive			Univariable			
Characteristic	Total household contacts, *N* = 1 428	Contact being a case, *N* = 57	Contact (non-case), *N* = 1 360	*N*	OR	95% CI	*p*-value
Exposure type				1 428			
Not sexual	921	12 (1.3%)	909 (99%)		Ref.	Ref.	
Sexual	320	38 (12%)	282 (88%)		10.2	5.42, 20.6	<0.001
Unknown	187	7 (3.7%)	180 (96%)		2.95	1.08, 7.43	0.025
Region				1 428			
London	634	18 (2.8%)	616 (97%)		Ref.	Ref.	
Not London	613	39 (6.4%)	574 (94%)		2.33	1.33, 4.20	0.004
Unknown	181	0 (0%)	181 (100%)				
Month of report				1 400			
July	533	24 (4.5%)	509 (95%)		Ref.	Ref.	
May	134	4 (3.0%)	130 (97%)		0.65	0.19, 1.72	0.4
June	449	15 (3.3%)	434 (97%)		0.73	0.37, 1.40	0.4
August	239	11 (4.6%)	228 (95%)		1.02	0.47, 2.08	>0.9
September	36	2 (5.6%)	34 (94%)		1.25	0.20, 4.45	0.8
October	1	0 (0%)	1 (100%)		0.00		>0.9
November	8	1 (13%)	7 (88%)		3.03	0.16, 18.0	0.3
Unknown	28	0	28				
Age (years)				1 428			
0–15	92	0 (0%)	92 (100%)		0.00		>0.9
16–25	112	5 (4.5%)	107 (96%)		0.77	0.23, 2.38	0.7
26–35	249	21 (8.4%)	228 (92%)		1.52	0.68, 3.74	0.3
36–45	202	21 (10%)	181 (90%)		1.91	0.85, 4.72	0.13
46–55	140	8 (5.7%)	132 (94%)		Ref.	Ref.	
56+	170	2 (1.2%)	168 (99%)		0.20	0.03, 0.80	0.042
Unknown	463	0 (0%)	463 (100%)				
Gender				1 428			
Male	972	51 (5.2%)	921 (95%)		Ref.	Ref.	
Female	430	6 (1.4%)	424 (99%)		0.26	0.10, 0.55	0.002
Unknown	26	0 (0%)	26 (100%)				
Overall	1 428	57 (4.0%)	1 371 (96%)				

### Secondary attack rate

The secondary attack rate among household contacts for England, Wales and Northern Ireland was 4% (57/1 428). A higher proportion of household sexual contacts (12%) went on to become a case compared to household contacts who did not report sexual contact (1.3%). Household sexual contacts were at 10 times increased odds (OR: 10, 95% CI:5.4, 521) of developing mpox compared to those with no sexual exposure (Table 2). Household contacts with unknown type of contact also had increased odds (OR: 3.0, 95% CI:1.1, 7.4) of becoming a case compared to those with no sexual exposure.

Contacts resident outside of London had increased odds of becoming a case compared to residents within London (OR: 2.3, 95% CI:1.3, 4.2). Female household contacts had lower odds of being diagnosed with mpox compared to male contacts (OR: 0.26, 95% CI:0.10, 0.55).

### Multivariable analysis

We found significant associations between sexual exposure, living outside of London and male gender with becoming a secondary case, among household contacts. Analysis was restricted to 1 224 contacts for whom region of residence and gender were known. When controlling for region of residence and gender, sexual contact was associated with the greatest increase in odds of becoming a secondary case compared to no sexual contact (adjusted OR: 11, 95% CI: 5.5–22). Household contacts for whom information on the type of exposure to the cases was unknown were also at increased risk of being a secondary case, at almost three times the odds compared to those who reported no sexual contact (adjusted OR: 2.8, 95% CI: 1.0–7.1). Household contacts outside of London were associated with 2.9 times increase in the odds of becoming a secondary case compared to those in London, after adjusting for type of contact and gender. Female contacts had a 59% decrease in odds of becoming a secondary case after controlling for sexual exposure and region (adjusted OR: 0.41, 95% CI: 0.15–0.95; Table 3).

**Table 3.** Multivariable analysis examining factors independently associated with a household contact becoming a secondary mpox case, England, Wales and Northern Ireland, 6 May to 24 November 2022

Characteristic	aOR^a^	95% CI^a^	*p*-value
Exposure type			
Not sexual	–	–	
Sexual	11	5.5, 22	<0.001
Unknown	2.8	1.0, 7.1	0.036
Region			
London	–	–	
Not London	2.9	1.6, 5.4	<0.001
Gender			
Male	–	–	
Female	0.41	0.15, 0.95	0.054

a aOR, adjusted odds ratio; CI, confidence interval.

## Discussion

Our study demonstrated a low secondary attack rate of 4% among household contacts of cases of mpox overall across the UK. This was similar to the 5% reported in previous studies in endemic West African countries and 4.7% in paediatric contacts in California, USA [4, 14]. To our knowledge, this was the first study to assess the risk of household transmission during the 2022 global outbreak.

We found transmission risk was strongly associated with sexual contact: household contacts who reported sexual exposure were at 11 times greater odds of infection when compared to those without sexual contact. This indicates that direct, prolonged contact with an infected body part(s) is a major driver of MPXV transmission.

The extremely low secondary attack rate in non-sexual household contacts, suggests that persons who come into contact with contaminated items or respiratory secretions within a household setting are potentially at lower risk of infection. Our study did not directly measure level and duration of contact between cases and contacts, but it can be expected that household contacts would be exposed to respiratory secretions or contaminated objects through shared spaces (toilets and kitchens) but not necessarily direct physical contact. Whilst respiratory infection and environmental contamination can occur, there is an absence of real-world epidemiological evidence to support it as a major driver of infection transmission [15].

Our findings are consistent with research identifying sexual contact as the main transmission mechanism during the global 2022 outbreak [16]. We found that female contacts were at lower risk of infection with MPXV, even after adjusting for sexual contact with the known case. As the 2022 outbreak primarily affected the GBMSM community, prolonged and direct non-sexual physical contact through bed sharing and/or care giving may have more frequently occurred between men.

We observed that household contacts who were resident outside of London were at greater chance of developing infection, after adjusting for sexual contact and gender. Reasons for this are unclear: although the epicentre of the UK outbreak was in London, many of those residing elsewhere reported travel to London, and most health promotion campaigns were UK-wide and targeted GBMSM groups. Guidance for follow-up of contacts, including testing and vaccination recommendations, were also consistent across the UK. Vaccination roll-out, however, was first implemented in London, and may have reduced the risk of contacts within London households becoming infected. When the outbreak was recognized, pre and post-exposure vaccination was targeted to those assessed at highest risk of infection, and/or risk of complications [17]. Vaccination was mostly through local sexual health services, and due to limited stockpiles initially, distribution had to be managed over time. Doses were first targeted in London, where the majority of the cases were resident. Up to the end of July 2022, 18 540 doses had been administered, with 16 411 in London (89%) [18]. Towards the end of the outbreak, as of 20 September 2022, a total of 45 049 administered doses of vaccine were recorded, of which 32 020 doses were given in London (71% of the total). The majority were administered as pre-exposure vaccination to gay, bisexual, and other men who have sex with men (40 426, 90% of doses up to 20 September 2022). A further 2 103 doses (4.7%) were given to healthcare workers managing monkeypox cases and 2 520 doses (5.6%) to close contacts of cases. A key limitation to our study was the lack of data on the vaccination status of contacts. A systematic review of studies in West African endemic settings found reduced secondary attack rates among vaccinated household contacts (to 0.98%, compared to 7.6% among unvaccinated) [4].

Another limitation of our study is its secondary use of an operational dataset, which may have introduced information bias. The study definitions prioritized specificity whilst trying to ensure adequate sensitivity. As a result, misclassification towards under-ascertainment of total contacts occurred. This was particularly apparent in defining sexual contact where insufficient information was available to classify many contacts within our dataset. The increased odds observed among household contacts with unknown exposure type, compared to not sexual, suggests it is likely this group included some people who had sexual contact with the index case. Furthermore, accurate onset dates were not always available to confirm directionality between index-secondary case pairs and validate that the index case was the most likely source of infection. This means we may have over-estimated the attack rate among household contacts by assuming household exposure to be the cause of infection when an alternative source may have been the true exposure. Household contacts may have additional risk exposures outside of the home, such as contact with other cases or attendance at venues where transmission may have been occurring, which may have resulted in infection. Household contacts were of similar age (where known) and gender profile to the cases and may have been flatmate or friendship groups rather than family households. Our findings may therefore not be generalisable to different household settings, and the missing data on age mean we may not have been able to accurately estimates the association.

Selection bias is likely present due to the stigma associated with sexual transmission, meaning cases may have been unwilling to declare contacts [19]. This is expected to have less of an impact on household contacts but may lower the number of household contacts declared, thus underestimating household size, and overestimating secondary attack rates, or mean that household contacts were wrongly reported as no or unknown sexual contact.

Our study demonstrated a low household secondary attack rate for mpox in England, Scotland, Wales, and Northern Ireland during the 2022 global outbreak, particularly for those with no sexual contact with the infectious case. This study is consistent with the evidence that behaviours, like sexual contact, with potential for prolonged contact with infected body parts, were a major driver of transmission of MPXV in the household setting during this outbreak. We recommend that when contact tracing for mpox, public health agencies should focus on gathering accurate and complete data on the nature of contact with the case and use this information to adjust their risk assessment based on whether direct physical contact with infected body parts has taken place. Contacts without direct physical contact could be managed as lower risk when considering the prioritization of testing, vaccination, isolation and other public health or infection control interventions, particularly when resources are scarce (such as limited doses of vaccine). Due to the high consequences of infection, however, careful risk assessment of household contacts, in particular regarding risk groups such as children and pregnant women, who were not well represented in this study, should continue to be part of public health response to mpox cases. We also recommend that routine collection of the vaccination status of mpox contacts is prioritized to monitor vaccine effectiveness at reducing the risk of transmission.

## Data Availability

The data that support these studies were collected as part of a public health response, are considered sensitive and not made publicly available. Reasonable requests for access to anonymised data will be considered by the authors on request.
